# 

**Published:** 1921-08-06

**Authors:** 


### Bradford Hospital Fund.

IT is satisfactory to learn that the Bradford Hospital and Convalescent Fund now possesses 70,000 subscribers, whose contributions average 2s. 8d. a head. During the past six months £1,200 more has been handed to the hospitals by means of the Fund than in any previous half-year. The total sum is £5,400. The West Riding Galas alone produced over £2,000 after all expenses had been paid, of which, however, one-third may have to be paid in entertainments tax. Presiding at the quarterly meeting of the Fund, Mr. Herbert Gill referred to the expense of St. Luke's Municipal Hospital, which, he said, had cost £92,400 to maintain till the end of March. He also mentioned the difficulties experienced in the completion of the Nurses' Home at Field House. Though the building had been finished, Mr. Gill said that plasterers were unobtainable, despite the fact that many are out of work. The workmen were walking about the streets. It was a shame; the Home ought to have been open by this time.

### Maternity Home Supervision.

Hammersmith Borough Council originally proposed the supervision of Parkside Maternity Home by a rota of doctors, but the Ministry of Health will not agree, expressing the opinion that the general supervision of a maternity home should be in the hands of one officer, and that satisfactory administration and supervision cannot be attained if the officer responsible is changed from week to week. Hence the Ministry is unable to approve of the Council's rota scheme, considering that the appointment of an assistant medical officer of health, whose duties would include the supervision of the home, would be the most economical way of securing the services which the borough requires. The Council's Health Committee nevertheless has carefully reviewed the whole matter, and is still of opinion, having regard to all the conditions now prevailing, that the medical supervision can satisfactorily be provided by the appointment of a rota of doctors as suggested, and that the consideration of the appointment of an assistant M.O.H. for this purpose should be delayed until a more favourable opportunity occurs in the future.

### View Day at the Great Northern Hospital.

Over two hundred and fifty supporters of the Great Northern Central Hospital availed themselves of the invitation issued by the Chairman, the Marquess of Northampton, and the Committee of Management to visit the institution on View Day, July 27. In the absence of the Marquess of Northampton, visitors were received in the Board Room of the Hospital by the Rt. Hon. H. J. Tennant, Vice-Chairman, who, in the course of a brief speech, said that the sum required to meet all their liabilities is £28,000, and that they are in urgent need of £15,000. The Committee, Mr. Tennant remarked, is responsible, in addition to the Central Hospital, for the upkeep of the Convalescent Home at Clacton, "Grovelands" Hospital of Recovery at Southgate, and the Royal Chest Hospital in the City Road. The wards were open for inspection and tea was provided in the nurses' garden.

August 6, 1921] [Price Twopence

### The Hospital

**RATES OF SUBSCRIPTION** (post free, payable in advance).

**Three months, 3/3; six months, 6/6; twelve months, 13/-. (Should the cost of printing and paper as well as increased postage, necessitate alteration of these rates, the period paid for will be reduced proportionately on unexpired subscriptions.) THE HOSPITAL "Index" to Volume LXIX., October 1920 to March 1921, is now ready, price 1/1 per copy, post free. Address The Manager, The Hospital, "The Hospital" Building, 28/29 Southampton Street, Strand, London, W.C. 2.**

